# Sympathetic-transduction in untreated hypertension

**DOI:** 10.1038/s41371-021-00578-5

**Published:** 2021-08-27

**Authors:** Matthew D. Kobetic, Amy E. Burchell, Laura E. K. Ratcliffe, Sandra Neumann, Zoe H. Adams, Regina Nolan, Angus K. Nightingale, Julian F. R. Paton, Emma C. Hart

**Affiliations:** 1 School of Physiology, Pharmacology, and Neuroscience, Clinical Research and Imaging Centre, University of Bristol, Bristol, UK; 2 Cardionomics Research Group, Bristol Heart Institute, University Hospitals Bristol NHS Foundation Trust, University of Bristol, Bristol, UK; 3 Department of Nephrology, Lister Hospital, East and North Hertfordshire NHS Trust, Hertfordshire, UK

**Keywords:** Risk factors, Hypertension

## Abstract

Transduction of muscle sympathetic nerve activity (MSNA) into vascular tone varies with age and sex. Older normotensive men have reduced sympathetic transduction so that a given level of MSNA causes less arteriole vasoconstriction. Whether sympathetic transduction is altered in hypertension (HTN) is not known. We investigated whether sympathetic transduction is impaired in untreated hypertensive men compared to normotensive controls. Eight untreated hypertensive men and 10 normotensive men (age 50 ± 15 years vs. 45 ± 12 years (mean ± SD); *p* = 0.19, body mass index (BMI) 24.7 ± 2.7 kg/m^2^ vs. 26.0 ± 4.2 kg/m^2^; *p* = 0.21) were recruited. MSNA was recorded from the peroneal nerve using microneurography; beat-to-beat blood pressure (BP; Finapres) and heart rate (ECG) were recorded simultaneously at rest for 10 min. Sympathetic-transduction was quantified using a previously described method. The relationship between MSNA burst area and subsequent diastolic BP was measured for each participant with the slope of the regression indicating sympathetic transduction. MSNA was higher in the hypertensive group compared to normotensives (73 ± 17 bursts/100 heartbeats vs. 49 ± 19 bursts/100 heart bursts; *p* = 0.007). Sympathetic-transduction was lower in the hypertensive versus normotensive group (0.04%/mmHg/s vs. 0.11%/mmHg/s, respectively; *R* = 0.622; *p* = 0.006). In summary, hypertensive men had lower sympathetic transduction compared to normotensive individuals suggesting that higher levels of MSNA are needed to cause the same level of vasoconstrictor tone.

## Introduction

The arrival of a sympathetic nerve action potential to the neurovascular junction initiates an intracellular cascade resulting in the release of noradrenaline and other co-transmitters [1]. Abluminal receptors on the vascular smooth muscle transduce this signal, usually resulting in vasoconstriction through the activation of adrenoreceptors [2]. This is a key mechanism by which the sympathetic nervous system (SNS) regulates arterial tone and hence arterial pressure in humans.

How effectively the vasculature can react to sympathetic neural activity (SNA) is dependent on the various mechanisms that transduce the signal from the postganglionic nerve terminals to the contractile responses of the vasculature. Other vasoactive molecules such as endothelin, purines, or nitric oxide can also modulate the efficacy of the SNA on vascular tone [3, 4]. The transduction of SNA into vascular tone is a major contributor to the ability of the SNS to control blood pressure (BP) [5, 6]. This process is sensitive to numerous other modulatory influences, such as how much neurotransmitter is released, the latency of neurotransmitter clearance from the synaptic cleft, and second messenger amplification within the vasculature [7]. The relative densities of α- and β- adrenoreceptors within the target area of the sympathetic bouton can result in vasoconstriction or vasodilatation depending on local receptor proportions [7]. Using different methods there have been multiple attempts to quantify the transduction of SNA over the last few decades [8–10]. For example, using an isometric handgrip to elevate BP and SNA from baseline [11]. Our group has created a novel analytical technique of measuring sympathetic transduction and has subsequently identified differences in the transduction of the SNA signal between both young and old individuals of both sexes [12].

In this study, we aimed to identify differences in sympathetic- transduction between normotensive men and untreated hypertensive men at rest. In short, transduction was quantified by measuring muscle sympathetic nerve activity (MSNA) and observing the subsequent effect on diastolic blood pressure (DBP). The method of measuring sympathetic transduction in this study has been previously described [12]. Surprisingly, there have been no studies to date that have examined differences in sympathetic transduction between normotensive and hypertensive individuals. We hypothesized that sympathetic transduction would be higher in untreated hypertensive individuals compared to normotensive individuals. Identifying differences in transduction could lead to a greater understanding of the SNS in hypertension and provide a new tool to predict the efficacy of current treatment options.

## Methods

### Participants

Following approval by the National Health Service ethics committee (11/SW/0207) and local R&D approval, we recruited 10 normotensive men and 8 untreated hypertensive men between the ages of 25 and 70 years. Men were recruited for this study because the balance between cardiac output, total peripheral resistance, and SNA is well understood and due to the fact that sympathetic transduction varies significantly in women [9]. All participants that took part in this study gave informed written consent to participate. The study was completed in full accordance with the Declaration of Helsinki.

Untreated hypertensive patients were defined as patients who are not currently taking any anti-hypertensive medications, an office BP recording of ≥140/90 mmHg, and ambulatory daytime BP ≥ 135/85 mmHg [13].

Normotensive patients were defined as having an average systolic blood pressure (SBP) during the daytime of <135 mmHg and DBP < 85 mmHg whilst not taking any medications, including antihypertensive agents. All the normotensive participants were healthy with no diagnosis of cardiovascular or pulmonary disease.

Participants were excluded from the study if they had any diagnosed cardiovascular disorders (excluding hypertension), such as cardiac electrical conduction abnormalities, heart failure or respiratory diseases such as chronic obstructive pulmonary disease (COPD). Other exclusion criteria included whether participants were currently suffering from any major illnesses such as cancer or inflammatory diseases, were currently taking steroids or nitrates, had a febrile illness within two weeks of the study date or had a body mass index (BMI) > 35 kg/m^2^. Intravenous drug users and individuals consuming more than 28 units of alcohol per week were also excluded.

### Screening

Participants were invited for a screening visit to establish suitability for the study. Clinic BP was recorded using an automated oscillatory sphygmomanometer (Omron, M6, Netherlands) while seated and in line with the 2018 European guidelines for BP measurement [13]. Urinalysis was used to screen for the presence of glycosuria, haematuria, and proteinuria, which might suggest underlying renal disease or diabetes. A 12-lead electrocardiogram (ECG) was performed and checked by a cardiologist to rule out any cardiac conduction abnormalities or ischaemia. If the study criteria were met, participants were sent home with an ambulatory BP monitor (ABPM) to be worn for 24 h (Spacelabs, 90217A, USA). The device inflated every 30 min during waking hours and every hour during sleeping hours [13]. Indexes of daytime blood pressure variability (BPV) were calculated as the SD of the daytime SBP and DBP, and the average real variability (ARV) of the SBP and DBP. This measure is the average of the absolute consecutive BP readings and is thought to be a better index of BPV than the SD because the SD reflects the dispersion of the BP reading around the mean, whereas the ARV reflects the actual variability between consecutive BP measurements [14]. ARV is also thought to be a better predictor of cardiovascular risk in people with hypertension versus the SD of daytime BP [15].

### Measurements

All of the measurements in this study took place in a clinical research unit at the Clinical Research and Imaging Centre (CRiC), Bristol, UK. The ambient temperature in the laboratory was controlled at 21 °C. The participants were asked to rest in the supine position at 30°. They were fitted with a continuous 3-lead ECG (BioAmp, Ad instruments) to measure heart rate and rhythm.

BP was measured continuously using a Finapres (Finometer® PRO, FMS) non-invasive BP monitor. The recording of BP using the Finapres was calibrated by using an automated sphygmomanometer on the same arm.

Multi-unit MSNA was recorded from the right peroneal nerve, laterally at the level of the head of the fibular as we have performed previously [9, 12]. The electrode in the nerve was aimed to target muscle sympathetic fascicles. A muscle sympathetic fascicle was identified when taps on the tibialis anterior muscle belly or passive muscle stretch evoked mechanoreceptive impulses, and no afferent neural response was evoked by skin or startle stimuli [16]. The recorded signal was amplified 80,000-fold, band-pass filtered (700–2000 Hz), rectified, and integrated (resistance-capacitance integrator circuit time constant 0.1 s) by a nerve traffic analyser.

### Data analysis

Data were sampled at 1000 Hz using a data acquisition system (LabChart, AD instruments) and stored on a laboratory computer (password protected) for later analysis. MSNA, heart rate, mean arterial pressure (MAP), SBP, and DBP were assessed as an average from the 10 min baseline. MSNA is expressed as burst incidence (bursts/100 heartbeats), burst frequency (bursts/min) and total activity (burst frequency multiplied by mean burst area; arbitrary units (AU)/min). The transduction was measured by quantifying the relationship between the MSNA burst area and subsequent DBP readings [12]. The individual bursts of MSNA activity were marked in an analysis suite (Spike 2 (v7), CED, UK) for the peak, start and end of each burst. Any drift present in the MSNA readings was removed by using a DC filter and the MSNA signal was normalized. For each participant, MSNA bursts were calibrated as a percentage with the amplitude of the largest burst represented as 100% and an area of baseline with no MSNA burst as 0%. We took each DBP reading and summed the MSNA burst area within two cardiac-cycle that preceded this DBP reading at a fixed lag. We search backwards from the DBP to allow us to include periods where there are no bursts in the neurogram. This fixed lag was calculated for each participant by plotting a waveform average (signal average) of MSNA events and DBP versus time and reading for the peak. The average lag (or delay) between a burst of SNA and the subsequent peak rise in DBP was 6–8 cardiac cycles. In four patients, it this was not possible to calculate the ‘lag’ as there was no clear peak in the rise of their BP after a burst had occurred, thus a fixed lag of 6–8 cardiac cycles was used as this was shown to be the average for participants. A window of two cardiac cycles at fixed lag for each individual was used to measure the area under the curve in the integrated MSNA neurogram (i.e., area under the curve within the 2 heartbeats). This was associated with the subsequent DBP (e.g., 6–8 cardiac cycles proceeding these heartbeats). We then plotted DBP vs. MSNA at the calculated lag to produce an XY scatter plot. The MSNA burst area was further binned into 1%/s bins with its associated DBP reading, averaged and put through a weighted linear regression. The slope of the regression was quantified as the MSNA transduction efficacy as previously described [12].

Time, frequency and non-linear indexes of short term heart rate variability (HRV) were measured using the LabChart HRV module (AD instruments, version 8) in accordance with the guidelines from the Task Force of the European Society of Cardiology and the North American Society for pacing electrophysiology [17]. Time-domain indexes included the root mean square of successive RR interval differences (rMSSD), the standard deviation of RR interval (SDRR), percentage of successive RR interval differences longer than 50 m (pRR50%) and the ratio of SDRR and the mean RR interval (CVRR). Frequency domain parameters of R–R intervals (via fast Fourier transform) included total power, very low frequency (VLF; 0–0.04 Hz), low frequency (LF; 0.04–0.15 Hz) and high frequency (HF; 0.15–0.45 Hz). Total power, VLF, LF and HF parameters were expressed in absolute values (ms^2^) and LF, HF and LF/HF ratios were also expressed as normalised units. Non-linear measures of HRV included the SD of the Poincare plot (SD1, SD2 and SD1/SD2 ratio). A Poincare plot was produced by plotting the RR interval against the subsequent RR interval. The SD1 is related to fast beat-to-beat variability whereas SD2 is linked to long-term RR interval variability and is influenced by both sympathetic and parasympathetic components. Indexes of parasympathetic function are thought to be rMSSD, SDRR, pNN50% and HF fluctuations in heart rate (in the 0.15–0.45 Hz power band). There is uncertainty regarding exactly what the LF component and LF/HF ratio represent physiologically [18]; with some suggesting they evaluate a sympathetic function, however, this has been debated in humans [19].

Finally, we calculated spontaneous indexes of sympathetic and cardiovagal baroreflex sensitivity (BRS). Spontaneous sympathetic baroreflex sensitivity (sBRS) was calculated by associating spontaneous fluctuations in DBP to the occurrence of bursts of MSNA. The analysis that we used has been termed “threshold analysis” and has been described in detail [20, 21]. In brief, DBP for each cardiac cycle (recorded during the baseline period) were grouped into BP bins of 1 mmHg. The % of heartbeats associated with a burst in each of these BP bins was calculated and associated with the mean DBP in the corresponding bin. The slope of the relationship between the mean DBP and mean MSNA for each DBP bin was calculated using linear regression. The slope of this relationship has been previously shown to agree with the BRS calculated during a modified Oxford baroreflex test [20]. The spontaneous cardiovagal BRS (cBRS) was assessed using the sequence technique [22]. Briefly, sequences of three or more successive heartbeats in which there were simultaneous increases or decreases in SBP and RR interval were selected using commercially available software (CardioSeries, version 2.4, Ribeirao Preto, SP, Brazil). Linear regression was applied to each of the sequences and an average regression slope was calculated for the sequences detected during each recording period. This slope represents the spontaneous cardiac BRS. Data regarding the slope of the cardiac baroreflex were averaged across falling and rising SBP.

### Statistical analysis

Data were statistically analysed using MatLab (The MathWorks, Natick, MA, USA). Data were checked for normality using the Shapiro–Wilk test and a two-tailed Student’s *T* test corrected for unequal variance was used to assess for differences in means. Pearson’s correlation coefficient was used to test for relationships between variables. Data are presented as mean ± standard deviation (SD). Alpha was set at 0.05.

## Results

### Participant demographics

**Fig. 1:**
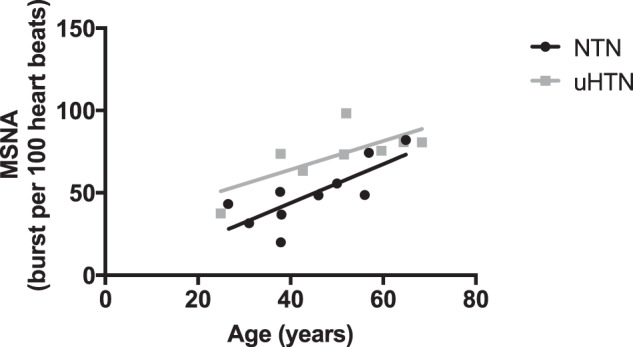
Correlation of age versus resting muscle sympathetic activity (MSNA), and sympathetic-vascular transduction. MSNA versus age (NTN; *r* = 0.61; *p* = 0.0072) or untreated hypertension (uHTN; *r* = 0.52; *p* = 0.042). There is no difference between the slopes of the correlation between age and MSNA (*p* = 0.52).

Table 1 shows participant demographics. BMI and age were similar between the hypertensive and normotensive groups. *Daytime* ABPM showed that DBP, SBP and MAP were higher in the untreated hypertensive men compared to normotensive controls. There was no difference in the BPV based on the SD of daytime SBP and DBP. Based on the ARV index of BPV, the hypertensive group had higher variability in the SBP vs the normotensives, but no difference in DBP. Clinic SBP, DBP and MAP were also higher in the hypertensive versus the control group. Ambulatory and clinic HR were similar between groups. In both groups, there was a significant correlation between age and MSNA as seen in Fig. 1 (bursts/100 heartbeats) (NTN *r* = 0.61; *P* = 0.0072, uHTN *r* = 0.52; *p* = 0.042).

**Table 1:** Demographics and baseline neural-haemodynamic variables in men with untreated hypertension and normotension.

	Normotensive men	Untreated hypertensive men	*P*-*V*alue
Age (years)	45 ± 12	50 ± 15	0.19
Height (m)	1.80 ± 0.07	1.76 ± 0.04	0.11
Weight (kg)	80.2 ± 10.3	81.4 ± 14.4	0.42
BMI (kg/m^2^)	24.7 ± 2.7	26.0 ± 4.2	0.21
*Daytime ABPM*			
SBP (mmHg)	124 ± 8	151 ± 25	0.003
DBP (mmHg)	80 ± 6	94 ± 18	0.019
MAP (mmHg)	95 ± 6	113 ± 20	0.008
HR (beats/min)	78 ± 10	74 ± 7	0.24
*ABPM BPV*			
SBP SD (mmHg)	13.3 ± 3.5	13.4 ± 4.6	0.75
DBP SD (mmHg)	10.0 ± 3.0	9.8 ± 1.3	0.83
SBP ARV (mmHg)	8.1 ± 1.1	12.3 ± 2.4	0.01
DBP ARV (mmHg)	7.6 ± 2.8	9.0 ± 1.7	0.28
*Clinic BP*			
SBP (mmHg)	125 ± 8	165 ± 32	<0.001
DBP (mmHg)	80 ± 8	98 ± 18	0.009
MAP (mmHg)	95 ± 9	120 ± 23	0.002
HR (beats/min)	66 ± 16	62 ± 7	0.22
MSNA (Bursts/100 Heartbeats)	49 ± 19	73 ± 17	0.007
MSNA (Bursts/min)	31 ± 7	45 ± 11	0.002
Total MSNA activity (AU/min)	372 ± 26	648 ± 35	0.001
sBRS (%/mmHg)	−3.08 ± 2.11	−2.50 ± 1.89	0.850
cBRS (ms/mmHg)			
All sequences	17.6 ± 6.2	16.8 ± 4.7	0.81
Up sequences	18.0 ± 6.3	15.22 ± 3.9	0.40
Down sequences	14.9 ± 4.3	19.5 ± 7.1	0.24

Data are shown as mean ± standard deviation. Data analysed using Student’s *T* Test.

*BMI* body mass index, *ABPM* ambulatory blood pressure monitoring, *MAP* mean arterial pressure, *HR* heart rate, *SBP* systolic blood pressure, *DBP* diastolic blood pressure, *BPV* blood pressure variability, *SD* standard deviation, *ARV* average real variability, *MSNA* muscle sympathetic nerve activity, *sBRS* sympathetic baroreflex sensitivity, *cBRS* cardiovagal BRS.

### MSNA and spontaneous BRS

MSNA was 48% higher in the untreated hypertensive group compared to the normotensive men (Table 1 for burst/100 heartbeats and bursts/min). There was a correlation between resting MSNA (bursts/100 heartbeats) and ambulatory daytime SBP (*r* = 0.54, *p* = 0.022) and MAP (*r* = 0.45, *p* = 0.048) when the groups were combined. There was no relationship between MSNA and ambulatory daytime DBP (*r* = 0.37, *p* = 0.013). MSNA expressed as bursts/min was correlated to daytime ambulatory SBP (*r* = 0.66, *p* = 0.003), DBP (*r* = 0.50, *p* = 0.04) and MAP (*r* = 0.58, *p* = 0.01). MSNA (bursts/100 heartbeats) was only correlated with clinic SBP (*r* = 0.44, *p* = 0.037) and not to clinic DBP (*r* = 0.18, *p* = 0.18) and MAP (*r* = 0.28, *p* = 0.26). MSNA (bursts/min) was significantly correlated to clinic SBP (*r* = 0.57; *p* = 0.01) and clinic MAP (*r* = 0.49; *p* = 0.04) but was not significantly correlated to clinic DBP (*r* = 0.40; *p* = 0.10). There were no differences in sBRS and cBRS between groups (Table 1).

### HRV

There were no differences in time- and frequency-domain or non-linear measures of HRV between hypertensive and normotensive groups (Table 2). Measures of HRV did however correlate with age (Table 3; SDNN, CVNN, total power (ms), LF (ms), HF (ms) and SD2). There was no correlation of HRV parameters to ambulatory daytime SBP or sympathetic- transduction (Table 3). Interestingly, there was a moderate correlation (*r* = 0.49) of sympathetic-transduction to LF HRV but did not reach *p* ≤ 0.05 (*p* = 0.056).

**Table 2:** Heart rate variability at rest in the normotensive and hypertensive participants.

	Normotensive men	Untreated hypertensive men	*P*-value
rMSSD (ms)	40.91 ± 15.4	34.7 ± 21.7	0.51
SDRR (ms)	54.1 ± 11.4	48.8 ± 22.7	0.85
pRR50 (%)	21.0 ± 17.8	14.0 ± 19.8	0.53
CVRR	0.06 ± 0.02	0.05 ± 0.02	0.52
Total power (ms^2^)	3180 ± 1404	2402 ± 2122	0.40
VLF power (ms^2^)	1088 ± 797	926 ± 689	0.67
LF power (ms^2^)	1168 ± 1020	747 ± 959	0.41
HF power (ms^2^)	885 ± 1125	695 ± 952	0.72
LF/HF	2.65 ± 2.74	1.91 ± 1.44	0.51
LF (nu)	56.1 ± 27.3	59.3 ± 16.0	0.78
HF (nu)	41.5 ± 25.4	39.4 ± 13.7	0.84
SD1 (ms)	29.0 ± 10.9	24.5 ± 15.3	0.51
SD2 (ms)	70.1 ± 15.6	64.1 ± 29.0	0.61
SD1/SD2	0.42 ± 0.16	0.37 ± 0.12	0.45

Values are mean ± SD. *P*-value from unpaired Students *t*-test.

*rMSSD* root mean square of successive *RR* interval differences, *SDRR* standard deviation of RR interval, *pRR50* percentage of successive RR interval differences longer than 50 ms, *CVRR* ratio of SDRR and the mean RR interval, *VLF* very low frequency (0–0.04 Hz), *LF* low frequency (0.04–0.15 Hz), *HF* high frequency (0.15–0.45 Hz), *SD* standard deviation of the Poincare plot.

**Table 3:** Pearson correlation coefficients to measure the relationship of age, SBP (ambulatory) and sympathetic transduction to heart rate variability parameters and sympathetic (sBRS) and cardiovagal (cvBRS) baroreflex sensitivity in all participants (hypertension and normotension grouped together).

	Age (*p*-value)	Sympathetic transduction (*p*-value)	Daytime ambulatory SBP (*p*-value)
rMSSD (ms)	−0.46 (*p* = 0.075)	0.10 (*p* = 0.685)	−0.19 (*p* = 0.487)
SDNN (ms)	**−0.66 (*p*** = **0.005)**	0.34 (*p* = 0.200)	−0.29 (*p* = 0.285)
pNN50 (%)	−0.42 (*p* = 0.105)	0.11 (*p* = 0.684)	−0.26 (*p* = 0.324)
CVRR	**−0.72 (*p*** = **0.002)**	0.46 (*p* = 0.075)	−0.24 (*p* = 0.378)
Total power (ms^2^)	**−0.67 (*p*** = **0.001)**	0.41 (*p* = 0.109)	−0.35 (*p* = 0.183)
VLF power (ms^2^)	−0.07 (*p* = 0.803)	0.16 (*p* = 0.545)	−0.22 (*p* = 0.424)
LF power (ms^2^)	**−0.64 (*p*** = **0.008)**	0.49 (*p* = 0.056)	−0.21 (*p* = 0.446)
HF power (ms^2^)	**−0.49 (*p*** = **0.050)**	0.14 (*p* = 0.594)	−0.25 (*p* = 0.349)
LF/HF	−0.16 (*p* = 0.562)	0.45 (*p* = 0.091)	−0.09 (*p* = 0.734)
LF (nu)	−0.01 (*p* = 0.803)	0.29 (*p* = 0.270)	0.09 (*p* = 0.727)
HF (nu)	0.07 (*p* = 0.794)	−0.30 *(p = *0.262)	−0.07 (*p* = 0.782)
SD1 (ms)	−0.46 (*p* = 0.075)	0.10 (*p* = 0.712)	−0.29 (*p* = 0.487)
SD2 (ms)	**−0.68 (*p*** = **0.004)**	0.38 (*p* = 0.149)	−0.29 (*p* = 0.276)
SD1/SD2	0.07 (*p* = 0.789)	−0.26 (*p* = 0.363)	0.06 (*p* = 0.577)
sBRS (%/mmHg)	0.35 (*p* = 0.211)	−0.29 (*p* = 0.331)	0.25 (*p* = 0.371)
cvBRS (ms/mmHg)	0.02 (*p* = 0.811)	−0.17 (*p* = 0.512)	−0.26 (*p* = 0.351)

See Table 2 for abbreviations. Values are Pearson’s *r* and respective *p*-value.

Significant correlation coefficients are in bold.

### Sympathetic-transduction

**Fig. 2:**
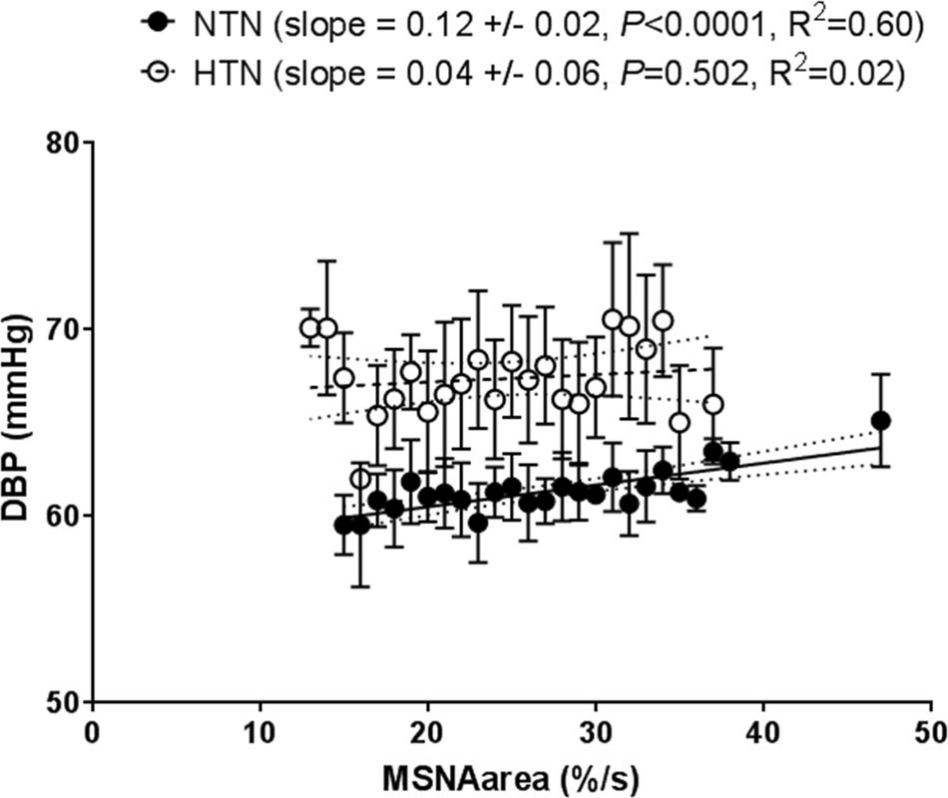
Example of data used to calculate sympathetic-vascular transduction in a hypertensive (HTN) and normotensive (NTN) male. For each DBP the MSNA burst area was measured (area under the curve) in a two cardiac cycle window at a fixed lag of 6–8 cardiac cycles preceding the DBP for both participants. This ‘window’ was moved across the whole baseline file, associating each DBP with an MSNA burst area. These data were represented as a scatter plot. MSNA burst area was then binned into 1%/s bins, and the corresponding DBP (mean ± SD) plotted. A weighted linear regression was then fitted to these data, the slope of which gave our measurement of transduction (units of mmHg (%/s)). MSNA muscle sympathetic nerve activity, DBP diastolic blood pressure.

**Fig. 3:**
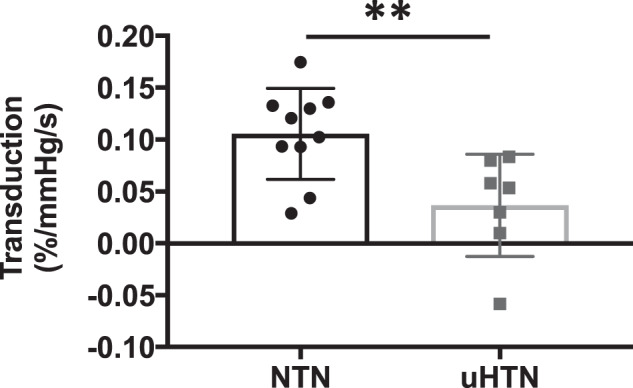
Sympathetic-vascular transduction in men with normotension (NTN) and untreated hypertension (uHTN). Mean ± standard deviation, **p = 0.0083.

An example of the linear regressions used to calculate sympathetic transduction is shown in Fig. 2, in a hypertensive and normotensive participant. After applying our method of quantifying sympathetic-transduction from MSNA and subsequent DBP readings, transduction was found to be lower in the hypertensive group compared to our normotensive controls (0.04 ± 0.05%/mmHg/s vs. 0.11 ± 0.04%/mmHg/s; *p* = 0.003; Fig. 3).

### Sympathetic-transduction vs. MSNA, age and BP

**Fig. 4:**
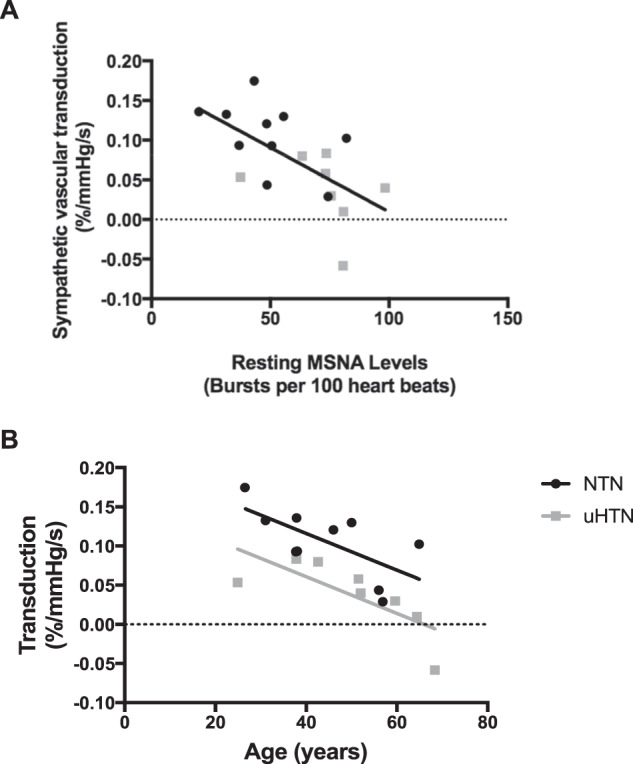
Correlations of sympathetic transduction to resting MSNA levels and age in both normotensive and untreated hypertensive men. **A** correlation of sympathetic-vascular transduction to resting muscle sympathetic activity (MSNA) in men with normotension (NTN; *r* = 0.48; *p* = 0.16) or untreated hypertension (uHTN; *r* = 0.33; *p* = 0.23). When the groups were combined there was an inverse correlation between the two variables (*r* = −0.622; *p* = 0.006). **B** Sympathetic transduction versus age (NTN; *r* = 0.44; *p* = 0.038) or untreated hypertension (uHTN; *r* = 0.56; *p* = 0.03). There is no difference between the slopes of the correlation between age and sympathetic-vascular transduction (*p* = 0.99).

Transduction was inversely correlated with increased resting MSNA (bursts per 100 heartbeats) in both groups individually as well as in the entire dataset (Fig. 4; *r* = 0.62; *p* = 0.006). The correlation was the same when MSNA measured in bursts per minute was used (*r* = 0.62; *p* = 0.006). Combining the data sets, we found that sympathetic-transduction was positively correlated with age (*r* = 0.67, *p* = 0.002), but not to BMI (*r* = 0.20, *p* = 0.42). When groups were considered separately, there was a correlation between age and transduction in both groups (NTN *r* = 0.44; *p* = 0.038, uHTN *r* = 0.56; *p* = 0.03). There was no difference between the slopes of the correlation between age and MSNA (*p* = 0.52), or between age and transduction (*p* = 0.99; Fig. 4).

The daytime SBP (*r* = −0.69; *p* = 0.002) and DBP (*r* = −0.68, *p* = 0.002) were inversely correlated with the level of sympathetic-transduction so that higher daytime SBP and DBP were linked to lower levels of sympathetic-transduction (or vice-versa). We also found that there was a strong inverse correlation between the ARV index of daytime SBP variability and sympathetic-transduction (*r* = −0.75, *p* = 0.001) so that higher transduction was linked to lower daytime BPV (or vice-versa). The ARV of DBP (*r* = −0.43, *p* = 0.075) or the SD of the SBP (*r* = −0.42, *p* = 0.091) and DBP (*r* = −0.29, *p* = 0.243) were not correlated with the level of sympathetic-transduction.

## Discussion

The main finding of this study is that men with untreated hypertension have a lower sympathetic transduction compared to normotensive men of the same age. The level of sympathetic transduction was inversely linked to the level of resting MSNA.

### MSNA

These results show that untreated hypertensive men have a significantly higher MSNA compared to normotensive men, supporting previous studies [9, 23–25]. The elevated MSNA is thought to be a factor involved in the development and maintenance of hypertension in humans [26]. Here we show that resting MSNA is positively related to daytime ABPM so that higher MSNA is associated with higher daytime BP. This supports previous data showing that MSNA is linked to the level of resting BP in middle-aged men and women [27]. MSNA was only moderately correlated with clinic SBP, but not to clinic DBP and MAP, suggesting that resting MSNA is better at predicting BP out of the clinic. The mechanism(s) underlying elevated sympathetic tone in people with hypertension are multi-factorial, but include activation of the renin-angiotensin-system [28], peripheral chemoreflex involvement [29], baroreflex dysfunction [30], chronic stress [31], poor organ perfusion [32], obesity as well as insulin and leptin levels [26, 33].

### Neurovascular transduction

We used a previously validated measure of sympathetic transduction [12] to estimate whether sympathetic- transduction is altered in men with untreated hypertension. This measure of transduction provides an estimate of how responsive the DBP is to beat changes in the MSNA area. Thus, the data suggest that the responsiveness of the DBP to a given increase in MSNA is lower in men with hypertension versus normotensive men. The results infer that in men with hypertension, a given increase in the level of MSNA causes less vasoconstriction versus that in men with normotension. Potentially, men with hypertension need a larger increase in MSNA to achieve the same change in DBP, but this needs to be assessed using interventions that increase MSNA. The data also suggest that men with untreated hypertension would also need a greater change in MSNA versus normotensive men to achieve the same fall in DBP. For example, the average sympathetic transduction slopes indicate that to achieve a 1 mmHg fall (or rise) in DBP, the area of the MSNA burst would need to decrease (or rise) by 8.3%/s in the normotensive men and 25%/s in the untreated hypertensive men. This assumes that the regression curve is linear (see Fig. 2 for example regressions). However, if we were to test across a whole range of MSNA and DBP, it is likely that the regression is sigmoidal.

There are several potential mechanisms that may explain the lower sympathetic transduction in patients with untreated hypertension. First, hypertension is associated with higher vasoconstrictor tone and total peripheral resistance [7]. It is possible that hypertensive patients have a tonic contraction of the smooth muscle which is close to the saturation point (i.e., no more force can be generated by the muscle by further depolarization), therefore the smooth muscle cannot contract further to increased sympathetic stimuli. Second, the vessel remodelling that occurs in hypertension [34] may mean that the vessels are less able to constrict in response to sympathetic stimuli. For example, fundamental changes that occur within smooth muscle cells in hypertension, such as increased collagen, may alter the mechanical function of the cell [35] reducing the degree of absolute contractility. Third, α-adrenoreceptor desensitisation due to chronic exposure to noradrenaline may occur [25]. This could involve uncoupling of the receptor from the G protein complex and the internalisation of the receptor. Along these lines, we show that the estimate of sympathetic-transduction is inversely linked to the level of MSNA. However, previous reports have shown that forearm vascular responses to noradrenaline are similar to normotensives in treated-hypertensive [36] as well as patients with hypertension who discontinued medication for 2 weeks before assessment [37], suggesting that vasoconstrictor responses to exogenous α-adrenergic agonists are similar in hypertension. However, other studies indicate that the response of forearm vascular resistance to α-adrenergic receptor agonist infusion is elevated [38, 39] or not changed in people with hypertension [40]. The disparity between these studies and ours may be explained by the techniques used. Here we show that dynamic responses to SNA may be blunted in hypertension, whereas intra-arterial infusion of an agonist and assessment of responses are completed over a longer time period and reflect ‘steady state’ responses of the vessels. In addition, the studies using exogenous administration of adrenergic agonists only consider post-synaptic mechanisms and do not allow an assessment of alterations in pre-synaptic modulation via autoreceptors on sympathetic fibres. Further studies will be needed to elucidate the true underlying mechanism for our novel observations.

Finally, despite a lower measure of sympathetic-transduction in hypertension, MSNA was directly linked to the level of ambulatory BP. Potentially, the overall higher level of MSNA supports a higher tonic vasoconstrictor tone despite the lower sympathetic transduction, which contributes to higher BP in hypertension. It is also possible that the tonically higher MSNA in hypertension contributes to the development of vascular stiffness (via remodelling of vascular smooth muscles cells [34]) so that high MSNA supports a higher BP.

### Limitations

In this study, 8 uncontrolled hypertensive men and 10 normotensive men were investigated. This is a small sample size. Our study sample was only in men, as sympathetic transduction has been shown to vary significantly in women [12]. Our sample was also not matched for age (on a case-control basis), however, as the mean age of both groups were similar it is likely that this is only of minor importance. Another potential limitation is that this study was unmatched for race or ethnic group, as it has been shown that vascular adrenergic constriction is increased in people with African Caribbean ethnicity [41]. Finally, we were unable to measure cardiac output during this study using valid techniques, thus we do not have measures of total peripheral resistance. Using changes in resistance in response to MSNA would have provided a further measure of sympathetic transduction into vasomotor tone.

### Implications

Hypertension has been shown to cost the NHS in excess of £2 billion per year. It is estimated that currently, 33% of adults worldwide have high BP [42]. Further to this, 60% of people being currently being treated for hypertension have BP controlled to within the normal range [42]. As a risk factor with such a profound burden on global health, increasing our knowledge and understanding of the pathogenesis of hypertension is vitally important. Our study has shown that untreated hypertensive men have lower sympathetic-vascular transduction than normotensive men. Thus, although MSNA is high, the vasoconstrictor effects of further increases (or dilator effects of decreases) in MSNA are blunted in untreated hypertensive males. This may offer a protective mechanism of preserving the vasculature against further increases in MSNA. However, it also suggests that when the MSNA reduces, there may be less of a decrease in DBP of vasoconstrictor tone. These findings also highlight the fact that when considering the role of the SNS in hypertension, more than just the level of SNA should be taken into account [43]. A richer understanding of how sympathetic-vascular transduction is implicated in hypertension may help us target anti-hypertensive medications more accurately as well as tailor our existing therapies more effectively. Future studies could examine how different classes of anti-hypertensive treatments affect sympathetic transduction both acutely but also chronically, especially given that many treatments do not reduce SNA despite a decrease in BP.

### Summary

#### What is known about topic

- The transduction of sympathetic nerve activity (SNA) into vascular tone is a major contributor to the sympathetic control blood pressure (BP).
- Using different methods there have been multiple attempts to quantify the transduction of SNA over the last few decades.
- Our group created a novel analytical technique of measuring sympathetic transduction, which confirmed differences in the transduction of the SNA into vascular tone between men and women.
- Since the vasoconstrictor response to a given level of SNA could impact BP control it is important to understand whether there is a difference in sympathetic transduction between people with hypertension versus normotensive controls.

#### What this study adds

- This study has examined differences in sympathetic transduction between normotensive and hypertensive males.
- The main finding of this study is that men with untreated hypertension have *lower* sympathetic transduction compared to normotensive men of the same age.
- The level of sympathetic transduction was inversely linked to the level of resting muscle sympathetic nerve activity (MSNA), suggesting that desensitization of the vasculature to MSNA is linked to elevated adrenergic stimuli.
- These findings suggest that men with hypertension need a higher level of SNA to cause increases in BP. Conversely, the data show that they also need a larger reduction in SNA to cause the same fall in BP.
